# The Interplay of Oxidative Stress and ROS Scavenging: Antioxidants as a Therapeutic Potential in Sepsis

**DOI:** 10.3390/vaccines10101575

**Published:** 2022-09-20

**Authors:** Sanni Kumar, Juhi Saxena, Vijay Kumar Srivastava, Sanket Kaushik, Himadri Singh, Khaled Abo-EL-Sooud, Mohamed M. Abdel-Daim, Anupam Jyoti, Rohit Saluja

**Affiliations:** 1 Amity Institute of Biotechnology, Amity University Rajasthan, Amity Education Valley, Kant Kalwar, NH-11C, Jaipur Delhi Highway, Jaipur 303002, Rajasthan, India; 2 Department of Biotechnology, University Institute of Biotechnology, Chandigarh University, S.A.S Nagar 140413, Punjab, India; 3 Department of Biochemistry, All India Institute of Medical Sciences (AIIMS), Bhopal 462020, Madhya Pradesh, India; 4 Pharmacology Department, Faculty of Veterinary Medicine, Cairo University, Giza 12211, Egypt; 5 Department of Pharmaceutical Sciences, Pharmacy Program, Batterjee Medical College, P.O. Box 6231, Jeddah 21442, Saudi Arabia; 6 Pharmacology Department, Faculty of Veterinary Medicine, Suez Canal University, Ismailia 41522, Egypt; 7 All India Institute of Medical Sciences (AIIMS), Bibinagar 508126, Telangana, India

**Keywords:** sepsis, oxidative stress, ROS, antioxidant

## Abstract

Oxidative stress resulting from the disproportion of oxidants and antioxidants contributes to both physiological and pathological conditions in sepsis. To combat this, the antioxidant defense system comes into the picture, which contributes to limiting the amount of reactive oxygen species (ROS) leading to the reduction of oxidative stress. However, a strong relationship has been found between scavengers of ROS and antioxidants in preclinical in vitro and in vivo models. ROS is widely believed to cause human pathology most specifically in sepsis, where a small increase in ROS levels activates signaling pathways to initiate biological processes. An inclusive understanding of the effects of ROS scavenging in cellular antioxidant signaling is essentially lacking in sepsis. This review compiles the mechanisms of ROS scavenging as well as oxidative damage in sepsis, as well as antioxidants as a potent therapeutic. Direct interaction between ROS and cellular pathways greatly affects sepsis, but such interaction does not provide the explanation behind diverse biological outcomes. Animal models of sepsis and a number of clinical trials with septic patients exploring the efficiency of antioxidants in sepsis are reviewed. In line with this, both enzymatic and non-enzymatic antioxidants were effective, and results from recent studies are promising. The usage of these potent antioxidants in sepsis patients would greatly impact the field of medicine.

## 1. Introduction

**Figure 1:**
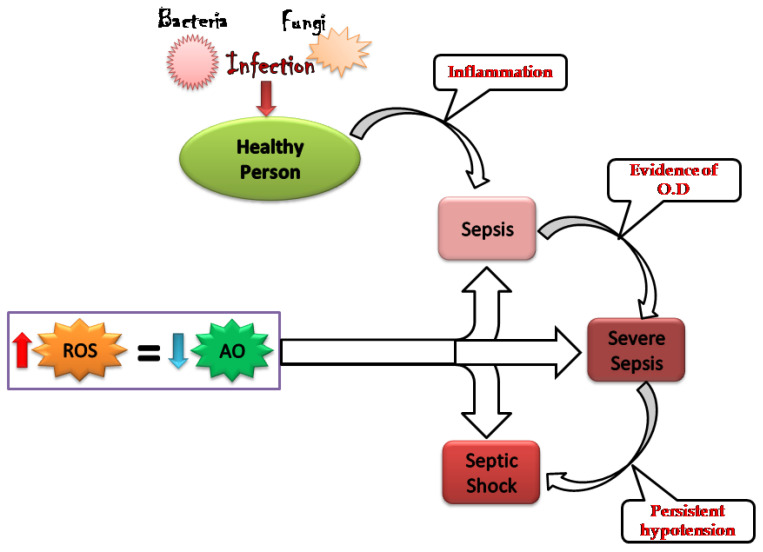
Schematic representation of the pathogenesis of sepsis with stages of severity with a view on oxidant and antioxidant levels.

Sepsis is an excessive or poorly regulated systemic inflammatory response that causes intravascular damage in the host [1]. Therefore, it remains an imperative cause of death worldwide. Systematic inflammatory response syndrome (SIRS) is a state of hyper-inflammation that also involves hypo- or hyperthermia, tachycardia, and tachypnea. Sepsis includes the symptoms of SIRS with presumed infection [2]. Not surprisingly, sepsis causes one or more severe complications such as renal dysfunction, cardiac dysfunction, coagulation, and hypotension. Together, they are known as called multiple organ dysfunctions, which lead to death [3]. According to recent surveys, it seems to be an increase in the incidence of sepsis, with mortality rates of 30–50% [4]. However, reported mortality shows considerable variation across the globe [5]. Microorganisms are responsible for sepsis (Figure 1) in which bacteria account for 62%, and the contribution of fungi is nearly 20%. Streptococcus pneumoniae and Pseudomonas aeruginosa are the most widespread microorganisms associated with sepsis as being isolated and more recurrent between old-aged patients [6].

The initial stage of sepsis involves hyper-activation of the innate immune response resulting in the disproportionate release of cytokines [7]. At this time, the patient has a compromised immune system, and their body turns out to be more inclined to secondary infections [8,9]. Infection and inflammation provoke local and systemic responses, including the hyper-activation of neutrophils. The pathogenesis of sepsis involves the dysfunction of endothelial and epithelial cells with the dysregulation of immune cells such as neutrophils and macrophages [9]. Cytotoxic effects of inflammatory mediators directly involve organ dysfunction with consistent hypoxia turning into multiple organ dysfunction [10].

In the present review, we have searched various public search engines such as PubMed and Google Scholar with the keywords sepsis, oxidative stress, ROS, and antioxidant. We have included research articles, case reports, and review articles falling between the years 2000 and 2022.

## 2. Oxidative Stress: Worsening the Pathology of Sepsis

The term oxidative stress represents an imbalance between oxidants (ROS), reactive nitrogen species (RNS), and antioxidants [11,12]. ROS notably contributes to the dysfunction of immune cells during sepsis. The concept that the highly reactive hydroxyl radical OH^.-^ could be generated from an interaction between superoxide O_2_^−^ and hydrogen peroxide H_2_O_2_ was proposed by Joseph Weiss [13]. Thereafter, it was recognized that the Haber–Weiss reaction O_2_^−^ + H_2_O_2_ --> HO + O_2_ + OH^−^ might provide a means to generate more toxic radicals. Even then, this often clearly indicates that disruption of free radical production or defenses at several various levels could cause harmful effects on cells [14]. Although the generation of HO, which is perhaps the most reactive oxygen species, is typically representative of an explicitly toxic phenomenon, it is through studies at this point that we could have progressed to a fuller insight of free radicals as both signaling molecules and toxic species. Oxidants, in general, encompass the initial species derived from oxygen and nitrogen, which reacts with most of the biomolecules and oxidizes them. Important ROS and RNS, as well as their source and catalyzed reactions, are enlisted in Table 1.

Free radicals including anion species (O_2_^−^, and OH^−^) and non-ionic species (H_2_O_2_, HOCl, and NO^.^) are the main culprits aggravating the pathogenesis of sepsis [21]. The key sites of ROS production in sepsis are activated neutrophils and macrophages. All through inflammation, these cells show signs of intestinal mucosa infiltration and release large amounts of reactive species [22]. Abundant production of ROS is associated with chronic inflammation in sepsis. Since we are in constant contact with oxygen, ROS is constantly produced in our body in controlled order, and their effect is counteracted by physiological antioxidant defense mechanisms. The defense system intercepts the ROS and repairs the damage that has already occurred by them [23]. Under normal conditions, the potentially harmful effect of ROS is successfully restrained by the defense mechanisms. Nitric oxide (NO) is protective of the vasculature and is an important neurotransmitter in the nervous system [24]. It is vital to the immune system and important for gene expression, signal transduction, and growth regulation [25]. NO derived from neutrophils and macrophages reacts spontaneously with O_2_^−^ to form ONOO^−^, a highly potent oxidant. Furthermore, the NADPH oxidase complex produces ROS through phagocytosis, acting as a vital factor for microbicidal activity [26]. Studies have shown that ROS can modulate immune signals, which results in affecting the pathogenesis of sepsis and causing damage to cells and organs [27,28]. When lipopolysaccharides (LPS) binds with Toll-like receptors (TLR)-4, it triggers the MyD88-dependent pathway, resulting in the activation of the nuclear factor kappa B (NF-κβ) pathway through the ROS-mediated inhibitor of nuclear factor κB kinase(IκκB) phosphorylation [29]. NF-κβ promotes the transcription of genes, such as NLR Family Pyrin Domain Containing 3 (NLRP3), Pro-IL-1β, and Pro-IL-18, which are essential for inflammation activation. These genes contain NF-κβ binding sites in their promoter region making it the most prominent and straight target of NF-κβ. IkkB-deficient macrophages exhibit uncontrolled activation of caspase 1 and enriched secretion of IL-1β upon LPS stimulation. TLR-4 induces NF-κβ-dependent expression of cytokines and chemokines [30]. DeLeo et al. have reported that LPS priming to neutrophils resulted in the activation of Nicotinamide adenine dinucleotide phosphate (NADPH) oxidase assembly by upregulation of the Rac2 protein [31].

In addition to direct involvement in cell death, ROS also plays a role as a secondary messenger in different signaling pathways associated with the inflammatory cells, and importantly, activation of NF-κβ. In the nucleus, NF-κβ binds to the promoter region and initiates the transcription of genes involved in inflammation [32]. It also upregulates cytokines (such as IL-2, IL-6 and TNF), cell adhesion molecule (vascular CAM-1) [33], growth factors (granulocyte colony-stimulating factor (GCSF) and macrophage colony-stimulating factor (MCSF) [34] and inducible nitric oxide synthase (iNOS) [35]. The involvement of ROS-mediated NF-κB activation leading to sepsis is well established [36,37]. Blackwell et al. have reported that LPS administration results in the activation of NF-κβ in several organs with an increase in cytokines such as TNF-α and IL-6 [38]. Bohrer et al. [39] compared increased NF-κβ activity with the Acute Physiology and Chronic Health Evaluation (APACHE II) score as an indicator of mortality in sepsis. Moreover, Arnalich et al. [40] have shown that NF-κβ activity was significantly elevated in non-survivors and had a direct correlation with the APACHE II score. Several studies evaluating the oxidant condition of critically ill and sepsis patients have been reported [41]. Lipid peroxidation products such as malondialdehyde (MDA) and 4-hydroxynonenal (4-HNE) are elevated in the serum of sepsis patients [42]. Furthermore, myeloperoxidase (MPO) activity is elevated in the lungs of patients with acute respiratory distress syndrome (ARDS) [43]. Disseminated intravascular coagulation (DIC) is a condition in which small clots develop in the bloodstream and is caused by RNS resulting in lipid peroxidation. Furthermore, dysregulation of selenium causes oxidative damage during endotoxemia and inflammation [44]. There are genes such as Interleukin-1 receptor-associated kinase 3 (IRAK3), Adrenomedullin (ADM), and arachidonate lipoxygenase 5 (ALOX5), whose expressions were significantly upregulated in sepsis patients. It shows their involvement in sepsis pathophysiology and indicates utilization as biomarkers or therapeutic targets [45,46]. Long non-coding RNA (lncRNA) MIAT has been shown to promote inflammation and oxidative damage in sepsis-induced cardiac injury via regulating miR-330-5p/TRAF6/NF-κβ axis [47].

**Figure 2:**
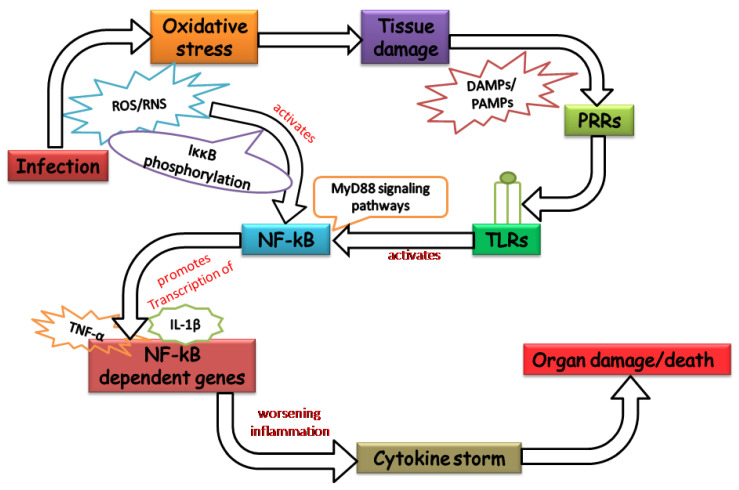
The mechanism by which the organ-damaging pathophysiology of sepsis is exacerbated by oxidants.

Oxidants cause a change in the redox state of the cell at the intracellular circumstances contributing to progression in inflammation and leading to organ dysfunction such as kidney dysfunction [48], liver dysfunction [49], and lung infection [50]. Emphasizing these pathophysiological mechanisms is important, but it has a limitation, as it depends on the pathogen and the patient in sepsis. The mechanism underlying ROS-mediated worsening of the pathology of sepsis leading to organ damage has been illustrated in Figure 2.

Mitochondria, the powerhouse of the cell, may sometimes turn into a foe for the cell due to leakage of an unpaired electron during the electron transfer chain resulting in the generation of O_2_^−^, an important contributor to the pathogenesis of sepsis [31,32,47]. Although this defective reduction accounts for less than 5% of the reactions that occur, it significantly reduces the respiratory capacity of affected tissues, leading to energy depletion (low level of ATP) and fatigue (high level of lactate) [33,34,35].

Organ dysfunction or organ failure is the worst outcome in patients suffering from severe sepsis. Apoptosis and necrosis are the major players mediating organ failure. Oxidative stress-mediated opening of mitochondrial permeability transition pore (MPTP), leading to diverse downstream pathways in both apoptosis and necrosis, results in cell death and ultimately organ failure. Cells with a low level of ATP are further affected by metabolic/chemical stresses and undergo necrosis [38,39]. During apoptosis, MPTP opening is associated with the efflux of Ca^2+^, loss of membrane potential, the release of pro-apoptotic proteins, excessive production of ROS, and the release of cytochrome c to the cytosol [51]. Mitochondria-derived ROS provokes the level of pro-inflammatory cytokines such as IL-1β, IL-6, and TNF-α, which further worsen the pathology of sepsis [52].

In addition to oxidative damage, nitrosative stress mediated by NO^.^ plays an important role in the pathogenesis of sepsis. Under an inflammatory condition, mitochondrial NOS present over the inner mitochondrial membrane generates NO in a larger quantity [53]. Being short-lived and highly unstable, NO reacts spontaneously with O_2_^−^ to form ONOO, which further inactivates complex IV of the mitochondrial respiratory chain [54,55,56]. A study involving septic rats demonstrated that NO-induced nitrosylation in thiol groups of complex I of the mitochondrial electron transfer chain results in energy depletion and is correlated with reduced levels of GSH [57]. NO has been shown to modulate the levels of key mitochondrial metabolites (succinate, and citrate), and subunit (Complex I) in an in vitro murine model of sepsis [58]. A positive correlation between blood level of NO and reduced mitochondrial activity leads to myocardial contractility [59]. During an LPS-induced model of sepsis in hepatocytes, an augmented level of NO-mt ROS signaling network hijacks the mitochondrial quality control system, further establishing an important role of NO and ROS in the pathogenesis of sepsis [60].

## 3. Antioxidants as a Potential Therapy for Sepsis

Oxygen-centered reactions are important for aerobic life, but uncontrolled ROS/RNS generation is lethal. To combat this, there are several antioxidants present endogenously and collectively known as the antioxidant defense system. Their role is to limit the amount of ROS/RNS with their defined distribution to assigned parts of the body. All the antioxidants exist in both reduced and oxidized forms. These antioxidants can be categorized into enzymatic and non-enzymatic groups (Table 2).

The enzymatic antioxidants mainly include superoxide dismutase, catalase, and glutathione peroxidase. These enzymes require cofactors for their activity such as zinc or manganese for superoxide dismutase, iron for catalase, and selenium for glutathione peroxidase. The non-enzymatic antioxidants include vitamins such as vitamin E, C, and A. Taking them all into our diet is essential, and they have potential effects on malnutrition. The non-enzymatic antioxidants also include hormones such as melatonin and synthetic compounds such as N-Acetylcysteine (NAC). Table 3 enlists different antioxidants investigated in sepsis and used in animal and clinical trials.

### 3.1. Superoxide Dismutase (SOD)

SOD is a metalloenzyme that catalyzes the partitioning of the superoxide anion to hydrogen peroxide [73]. This hydrogen peroxide is later metabolized into the water by glutathione peroxidase and catalase. Various forms of SOD exist. In humans, SOD takes the form of cytosolic copper-zinc containing SOD (Cu-Zn-SOD), manganese-requiring mitochondrial SOD (Mn-SOD), and an extracellular SOD (EC-SOD) [74]. Nickel-containing SOD (Ni-SOD) is present in prokaryotes, and iron-containing SOD (Fe-SOD) is present in bacteria as well as in plants [75]. SOD enzyme has the highest activity levels in organs such as the liver, kidney, spleen, and adrenal gland [76]. In the mitochondria, O_2_^−^ is generated, which is later dismutation to H_2_O_2_ by both Cu-Zn-SOD and Mn-SOD present in the mitochondrial matrix [50]. It is also reported that gastric ulcers are caused by low SOD activity. Supplementation of a high concentration of SOD helped in curing ulcers in patients [77]. ROS and protease enzymes are released due to hyper-activation of neutrophils, which damage normal tissue, and later cytokine release further worsens the inflammation [76]. The endothelial cells are activated by superoxide anions and increase neutrophil penetration by upregulating adhesion molecules (ICAM-1) [78]. An animal study conducted by Salvemini et al. [79] showed that the administration of SOD mimetics attenuated the superoxide and peroxynitrite-mediated intestinal damage induced by endotoxin. Later, Ghio et al. [52] experimented and confirmed that overexpression of EC-SOD decreases lung injury. Investigators have reported that SOD mimetics of the MnII complexes of Pytren4Q (Mn-L1) and Pytren2Q (Mn-L2) have immense anti-oxidant activity in SOD-deficient bacteria and yeast [80,81]. Serena et al. [82] have shown that Mn-L1, a SOD mimic, effectively protects human-cultured THP-1 macrophages and total mice from the inflammatory consequence formed by LPS. An in vitro study conducted by Coleman et al. [83] has shown the positive effect of SOD mimetic (BuOE), as it protects the loss of mitochondria. These responses illustrate the importance of SOD antioxidant activity in promoting health.

### 3.2. Catalase

Catalase is one of the most abundant enzymes of mitochondria and peroxisomes in mammals [84] in humans, and it is found with high and low activity in the liver, kidney, and connective tissues. It dismutases H_2_O_2_ to H_2_O and O_2_. When glutathione peroxidase concentrations are low, catalase catalyzes the H_2_O_2_ generated during cellular metabolism [75]. Transgenic mice with low levels of catalase have shown normal development, but they are more sensitive to oxidative damage [84,85]. An in vivo study conducted by Siwale et al. [86] showed that after endotoxin stimulation, microencapsulated (MC) catalase inhibited H_2_O_2_ and TNF, resulting in improved survival. LPS stimulation causes platelet aggregative dysfunction, and an in vivo study performed by Dong et al. [87] reports the role of catalase in the prevention of platelet dysfunction. In one study, it has been shown that when NO level increases, it causes endotoxic shock in most of the organs such as the lungs, liver, and kidneys. After the SOD-CHS-CAT conjugate was administered, it showed a protective effect on renal and hepatic function [88].

### 3.3. Glutathione

Glutathione reductase (GR) reduces oxidized glutathione disulfide (GSSG) to glutathione (GSH) [89]. GSH comprises more than 90% of the non-protein with reduced thiols in cells. The GR enzyme is a flavoprotein disulfide oxidoreductase where each monomer subunit consists of four domains: (i) FAD-binding domain, (ii) NADPH-binding domain, (iii) central domain, and (iv) interface domain. The catalytic activity exists between the interface domains of the dimer [90]. The GR enzyme preserves RBCs, hemoglobin, and cell membranes from oxidative damage by inducing GSH [91]. Riboflavin deficiency leads to abridging the GR activity in cancer [92], specifically in colon cancer, elevated GSH levels are observed due to drug resistance [93]. Experimental data investigate that the protein concentration in cysteine amino acids shows an effective cysteine delivery system for GSH replenishment during the immune response [94]. A decrease in antioxidant enzyme GSH levels demonstrates the role of oxidative mechanisms in sepsis-induced tissue damage [95]. These findings are similar to the result and conclusion of the study performed by Berger et al. [53]. Combined supplementation of L-glutamine and L-alanine (GLN + ALA) have shown a positive effect in reducing oxidative damage as well as endotoxin-induced inflammatory response in mice [54]. Zong and Zhang [55] showed that inhibition of GSH synthesis by buthionine sulphoximine significantly suppressed the AMF-induced inhibitory effect on oxidative damage in the development of acute lung injury (ALI) in septic rats. In a recent study, it has been seen that S-Nitrosoglutathione (GSNO) shows beneficial effects against LPS-induced acute kidney injury (AKI). It shows antioxidant effects for the treatment of sepsis-induced AKI [96]. In another experiment, GSNO shows protection against LPS-induced epithelial barrier injury in rats [56]. Clinical trials of GR inhibitors should be focused precisely to understand the detoxification pathway of GST that will help in establishing the therapeutic target for inflammatory diseases.

### 3.4. Vitamin C

Ascorbic acid (AA) is the redox form of vitamin C and acts as a natural antioxidant. In sepsis, the circulating concentration of ascorbic acid is markedly reduced. In several animal studies, a high dose of AA is defensive by reducing the deleterious effects of oxidative damage in sepsis [97,98]. Vitamin C is a potent electron donor, which reacts with O_2_^−^ and has a protective role against the oxidative damage generated within leukocytes [99]. It also plays a role as a modulator for essential biological pathways of the normal metabolism of immune cells [100]. As an antioxidant, it hinders the activation of NF-κβ formed by endotoxin, which results in the attenuation of TNF-α [101]. Victor et al. [102] in their study performed using peritoneal macrophages from BALB/c mice suffering lethal endotoxic shock, concluded that vitamin C supplementation scavenges the free radicals hence reducing endotoxin shock severity. Later, Armour et al. [103] showed in their studies that it modulates the functions of lymphocytes in septic rats. This study also interrupted H_2_O_2_ injury to cultured microvascular endothelial cells. Rojas et al. [104] demonstrated that guinea pigs do not unify their vitamin C like humans; as endotoxin was administrated, it caused depletion of vitamin C and favored oxidative damage. Many studies show the depletion of vitamin C in patients with sepsis [105,106]. Carcamo et al. [107] reported the mechanism of vitamin C in the suppression of NF-κβ activation by inhibiting TNF-α-induced activation of NIK and IKKh kinases independent of p38 MAPK. Wu et al. [108] also identified that iNOS expression may be inhibited with the supplementation of vitamin C, which leads to reduced oxidant levels in the septic muscle. Borrelli et al. [109] reported that plasma AA in patients with multi-organ failure was significantly lower, whereas a similar result was obtained, and the concentrations were inversely correlated with increased lipid peroxides [105]. A study was conducted on critical patients in the ICU by Long et al. [110], and thereafter, he concluded that during trauma and infection, levels of AA in plasma are exceptionally low. When a high dose of vitamin C was infused intravenously into the patient with acute respiratory distress syndrome (ARDS), rapid resolution of lung injury has been seen [111]. A specific trial in major burn patients was performed where results showed that AA supplementation in high doses was able to diminish the capillary leak and the volume of fluid required for hemodynamic stabilization [112]. In an animal study, the antioxidant mechanism of AA and its involvement were directly associated with and have been accepted in burned sheep [113]. An in vivo study showed that Vitamin C-deficient mice were more inclined to sepsis-induced multiple organ dysfunction. After the mixing of Vitamin C, physiological functions were normalized, which attenuated the development of multiple organ dysfunction in sepsis [111,114]. In particular, Mohammed et al. [115] demonstrated that low levels of Vitamin C are directly correlated with the delay in the timing of the resolution of inflammation. These results suggest that the role of vitamin C as an antioxidant influences the inhibition of NF-κβ activation, resulting in less organ dysfunction.

### 3.5. Vitamin E

Vitamin E is an important antioxidant found in abundance, having a major potent role as a protector for cell membranes while protecting them from lipid peroxidation. α-tocopherol is a biologically active form of vitamin E [116]. In mammalian cellular membranes, vitamin E is a major chain-breaking antioxidant [117]. It has a very unique ability, where it limits oxidation in the bi-lipid cell membrane; due to this feature, it has several additional biologically imperative effects, most importantly, inhibition of protein kinase C (PKC) [118]. Many investigators in their studies have reported that vitamin E concentration was very low due to oxidative damage in patients with sepsis [119,120]. The ability of α-tocopherol to act as a pro- or antioxidant depends on the extent of α-tocopherol available to scavenge ROS [121]. It is also defensive in decreasing the effects of oxidative damage in sepsis shown in the study of animals. Declined mortality in guinea pigs infused with live bacteria has been observed due to a high dose of vitamin E [122]. In human trials, when volunteers were supplemented with Vitamin E, they had considerably suppressed responses to LPS acting as a potent immuno-modulator to hinder the activation of inflammatory cells [123,124]. In an in vivo study, vitamin E has been shown to have antioxidant properties, which prevented sepsis-induced changes in lung tissue [125]. A synthetic vitamin E derivative (E-Ant-S-GS) showed prominent anti-inflammatory actions with the protection of organ effects in a rat model of sepsis [126]. Another investigator used vitamin E derivative (ETS-GS), and they reported that it inhibits the secretion of cytokines in a cecal ligation and puncture (CLP)-induced sepsis rat model, resulting in reduced organ dysfunction [126]. These studies suggest that it can be used as a potential clinical therapeutic agent against systemic inflammation.

### 3.6. Vitamin A

Vitamin A is mostly referred to as carotenoids and it is mostly present in citrus fruits as well as green leafy vegetables. Carotenoids are found in both food and the body in different forms such as carotene α, β, and lycopene [127]. It has antioxidant properties that mostly depend on retinol-binding proteins and other endogenous antioxidants in vivo [128]. Animal studies have been conducted, and it was found that β-carotene has a role in the suppression of lipid peroxidation in mouse models [129]. The role of β-carotene as an anti-infective agent has been well established [130]. The underlying mechanism in the reduction of infection may be through modification of epithelial integrity and function with non-specific immunity of the host. Cox et al. [131] indicated through their study that the mutual effects of pregnancy and vitamin A deficiency cause suppression of pro-inflammatory type 1 immune responses. It was improved using low-dose vitamin A supplementation. A study has shown that β-carotene and lycopene can lower ROS production, hence attenuating inflammation [132]. Jang et al. [133] reported that β-carotene reduced the expression of targets of NF-κβ, including iNOS and COX-2, by 90% in bacterially infected gastric adenocarcinoma (AGS) cells and decreases the secretion of NO. Supplementation of vitamin A was able to decrease the incidence of bronchopulmonary dysplasia in low birth weight infants. A prospective evaluation has shown the positive effect of vitamin A in reducing the incidence of chronic lung disease [134]. The immune changing role of both vitamin A and D throughout the infection was monitored using a complete RNA sequencing-based methodology, whereas both vitamins showed a positive response against the pathogen-induced responses [135].

### 3.7. N-Acetylcysteine (NAC)

It has antioxidant properties and contains a thiol group. It is a major antioxidant because it is a source of glutathione groups in cells. It is a precursor of cysteine, which makes it a potent factor to limit the rate of formation of glutathione (GSH). It has a thiol group and because of this, it ‘directly’ scavenges reactive species [136]. Rank et al. [137] conducted studies in humans and demonstrated that the administration of NAC can significantly increase hepatosplanchnic blood flow attributed to the increase in the cardiac index. NAC has been shown to inhibit pro-inflammatory transcription factors AP-1 and NF-κβ, hence possessing anti-inflammatory properties [138]. These transcription factors are induced in response to oxidative stress, supporting the argument that the anti-inflammatory properties of NAC are due to its mechanism of action as an antioxidant [139]. Furthermore, Emet et al. [140] have demonstrated in their studies that NAC does not influence outcomes with the level of cytokines. Sometimes, due to NAC, sepsis-induced organ failure was even aggravated [141]. A study indicated that NAC has a preventative role in LPS-induced obstruction by reducing iNOS expression through peroxidation of the lipid in liver and renal tissue [142]. NAC administrations on critical patients with sepsis were studied, and investigators have found that a high dose of NAC has a role in increasing immunity during mechanical ventilation [143]. Plasmodium genus causes infection and can contribute to oxidative damage and lead to malaria. Supplementation of NAC decreased parasitemia and oxidative damage in a rat model, which has been reported by Quadros Gomes et al. [144]. The inclusion of NAC in fluid rejuvenation may enhance renal oxygenation, which further reduces vascular dysfunction. It is also involved in the decrease in renal NO levels, which shows a beneficial effect in acute kidney injury [145]. Furthermore, there was a meta-analysis performed by Visvanathan et al., where they had questioned the assurance and convenience of intravenous NAC as adjuvant therapy in sepsis. These investigators also claim that NAC is ineffective in reducing the mortality inpatient population and is very unsafe when administered later than 24 h after the commencement of symptoms [146]. A therapeutic strategy combining the microvascular effects of fluids and NAC may be critical to averting sepsis-induced AKI.

### 3.8. Melatonin

**Figure 3:**
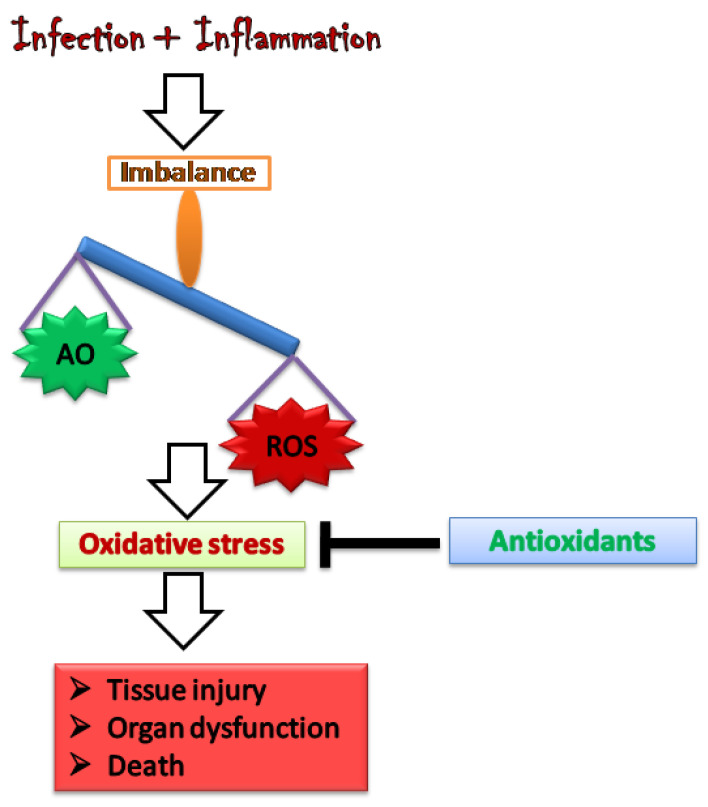
ROS: to maintain their functions and to avoid the negative effects of excessive ROS generation, immune cells require optimal amounts of antioxidant defenses. This figure shows antioxidants’ general protective mechanism in sepsis.

Melatonin is the hormone product of the pineal gland, involved in important physiologic functions. It is also synthesized by human PMNs, with multiple immune-modulatory effects [147]. It acts as a free radical scavenger, and it is believed to directly detoxify ROS via electron donation, which includes most importantly the hydroxyl radical and hydrogen peroxide [148]. It also detoxifies other oxidants such as nitric oxide, singlet oxygen, and peroxynitrous acid [149,150]. Melatonin scavenges the oxidants; during sepsis, there is an induction of iNOS yielding huge amounts of NO˙ that severely contribute to mitochondrial dysfunction [70]. It consistently inhibits the expression and activity of iNOS, which directly relates to its efficacy in preventing mitochondrial dysfunction during sepsis [151]. Escames G et al. [152] in their studies have shown that melatonin with its protective effect has counteracted the effect of LPS on respiratory complex I and iNOS activity in the liver and lung of rats. Ozkok et al. [153] have studied the effect of melatonin on the skeletal muscle of rats with LPS-induced endotoxemia. According to the histopathologic findings, they concluded that melatonin reduced myopathic changes in the LPS group. Melatonin is an anti-oxidant, and it has been shown to scavenge different types of free radicals in body fluids and cells [154,155]. Furthermore, melatonin activates anti-oxidant defense to reduce the amount of ROS leading to improving oxidative-related pathologies such as hypertension and brain diseases, for example, Huntington’s and Parkinson’s disease [149,156]. An experimental and clinical study has shown that melatonin is useful in fighting against sepsis and septic injury, the mechanism involved is the anti-oxidative and anti-inflammatory actions of melatonin [157]. Melatonin administration improved the clinical condition of neonatal sepsis patients. After some more clinical research, it was found that it could be used as a therapeutic target for the same [158]. The conceptual approach to the use of antioxidants for the protection against ROS-mediated organ damage in sepsis is depicted in Figure 3.

### 3.9. Antioxidants Protecting Mitochondria

Being that mitochondrial dysfunction leads to oxidative damage and that hyper ROS generation is an important contributor to the pathogenesis of sepsis, antioxidants targeting mitochondrial dysfunction pathways could improve a patient’s survival by counteracting the excessively produced ROS [33]. Because of a higher negative potential present in mitochondria, antioxidants tagged with lipophilic cations are an attractive approach to increase their penetration and retention inside mitochondria [111]. Several antioxidants have been used to ameliorate mitochondrial function by preventing oxidative damage in sepsis (Table 3). MitoQ, a unique formulation of antioxidant CoQ10 conjugated with a triphenylphosphonium (TPP) cation, quenches harmful free radicals in the mitochondria before their efflux and damage to the cells. MitoQ has been shown to protect mitochondria from oxidative damage by lowering the level of ROS, preserving membrane potential, suppressing the release of proinflammatory cytokines, and increasing the production of IL-10, an anti-inflammatory cytokine in the endothelial cell model of sepsis [175].

A study involving an LPS-induced sepsis model in mice demonstrated that MitoQ supplementation results in attenuation of plasma diamine oxidase, D-lactate, intestinal oxidative as well as nitrosative stress, and pro-inflammatory cytokines, and it augmented the level of intestinal SOD and GSH. MitoQ exerted this antioxidant and anti-inflammatory effect by activating the nuclear factor E2-related factor 2 (Nrf2) signaling pathway and its downstream antioxidant genes, including HO-1, NQO-1, and GCLM [183]. SkQR1, a complex formed between a plant quinone plastoquinone and TPP, inhibits TNF-induced endothelial permeability under in vitro conditions. SkQR1 exerts a mitochondria protective role by attenuating caspase 3 activations, β-catenin cleavage, and matrix metalloprotease 9 (MMP 9)-dependent shedding of transmembrane proteins [184]. RNA level molecules also ameliorate sepsis-induced mitochondrial dysfunction. In this context, H19, a type of lnc RNA, inhibits the sepsis-induced inflammatory response and mitochondrial dysfunction via modulation of the miR-93-5p/SORBS2 axis in LPS-treated cardiomyocytes [185]. SS31, a novel cell-permeable antioxidant peptide, known to be located in the inner mitochondrial membrane, has shown to be cardioprotective mediated by improving the oxidant/antioxidant balance and mitochondrial function linked to NF-κβ in LPS-induced cardiac damage in mice [181].

## 4. Failures and Risks of Antioxidants

Some earlier research studies indicate that supplemental antioxidants cannot diminish the risks for some diseases and can even play an opposite role because the antioxidant may not be involved in metabolism or may be a pro-oxidant in vivo [186]. Another reason for the failure of clinical assessments of antioxidant therapies is the use of antioxidants in the late stages of diseases, as in the case of atherosclerotic adult individuals. Thus, the selection of different stages of disease progression has been more probable to reveal the response from antioxidant remedies [187]. The addition of excess vitamin E to lipid emulsions can accelerate lipid peroxidation in vitro, even though vitamin E inhibits lipid peroxidation in normal conditions [188]. In vivo studies also showed that the administration of vitamin E raised plasma lipid peroxidation metabolite levels in smokers on a high-fat diet and induced fatty livers in rats that were maintained on an ethanolic high-fat diet [189]. A high intake of vitamin E in adults is generally thought to have only minimal toxicity, but blood coagulation can be affected by interfering with the action of vitamin K. Additionally, high doses of vitamin E might increase the overall mortality in cardiovascular disease and cancer patients in a dose-dependent manner [190]. Latent toxicity via increasing the linked oxidized low-density lipoprotein that is an important biomarker of oxidative damage after high dosages of vitamin E supplements with a high risk of cardiovascular diseases [191]. There are no significant differences that were detected in hemodialytic patients after massive supplementation of vitamin C in thiobarbituric acid reactive substances (TBARS) and lipoperoxides levels; consequently, ascorbic acid failed to prevent steady-state levels of lipid peroxidation [192].

Furthermore, several studies indicated that polyphenols decreased TBARS, and F2-isoprostanes, (8-iso-PGF2α, where PGF is prostaglandin) are produced by nonenzymatic oxidation of arachidonic acid by reactive oxygen species including free radicals [193]. F2-Isoprostane is the most commonly used isoprostane marker of lipid peroxidation to evaluate in vivo oxidative damage [194]. In this respect, some studies proposed that natural antioxidants that are rich in polyphenol content may prevent the oxidation of LDL in mildly hyper-cholesterolemic patients [195]. The water solubility of flavonoids may play a major role in therapeutic efficacy, as aglycones of low solubility flavonoids had lower absorption in the intestine, and poor availability was obtained. Therefore, partly synthetic water-soluble flavonoids were developed as hydroxyl-ethylrutosides and inositol-2-phosphatequercetin, for the treatment of hypertension and hemorrhage [196]. However, the in vivo antioxidant efficacy of flavonoids is less documented, and their pro-oxidant properties have been essentially illustrated. Flavonoids with pro-oxidant properties induced oxidative damage by reacting with lipoproteins and nucleoproteins [197]. People should recognize that excessive consumption of a natural supplement or food can be dangerous, and they may not attribute their side effects and symptoms to natural compounds [198]. The most recent literature has been focused on the advantageous activities of flavonoids, but few reports referred to how excessive consumption can result in significant health problems [199]. Therefore, further studies are required to identify conditions of antioxidants that are converting into pro-oxidants and their pathways as metabolic components. Additionally, there should be further clinical and experimental analyses to reveal diseases that are suitable for antioxidant therapy and how antioxidant intake can maintain health.

## 5. Future Perspectives

The involvement of the ROS-sensitive signaling pathways suggests that therapeutic targeting of oxidative damage with redox-modulating agents to prevent organ damage should be beneficial when treating sepsis. Targeted therapy of sepsis without side effects is the need of the hour. Nanoparticle-mediated drug delivery is gaining importance because of its high specificity and selectivity over other drug-delivery systems. Currently, few nano-conjugated antioxidants have been evaluated for the treatment of ROS-mediated organ toxicity in sepsis. In view of this, extensive research needs to be performed utilizing nano-conjugated redox modulating agents with improved pharmacokinetics and pharmacodynamics for the prevention of organ damage during sepsis.

In silico prediction of the antioxidant potential of key proteins is an excellent approach, which facilitates in reducing the number of possible candidates for further authentication by wet-lab experiments. Various computational methods based on machine learning algorithms have been developed using protein sequences to predict the antioxidant nature of proteins (Table 4). These provide useful insights into the study of antioxidant proteins and help researchers understand the role of different properties of antioxidants in their antioxidative activities. Despite this, further studies are required to develop better and more effective tools using advanced algorithms to enhance specificity, sensitivity, and prediction accuracy.

## 6. Conclusions

The significance of the damage inflicted upon biological systems by ROS cannot be overruled, as they have been implicated in sepsis. The dual nature of these species with their benign and destructive characteristics signifies the complexities of their specific functioning in sepsis. Evidence on the harmful effects of oxidative damage in pathophysiological mechanisms of sepsis is compelling. The use of known antioxidants to boost the defense system could be used to reinstitute this imbalance and should provide benefits in impairing and curtailing the harshness of sepsis. At the same time, undertaking the identification of novel and more potent antioxidants as a therapeutic strategy should continue. This review hopes to stimulate researchers to become more involved in this area and carry out the novel investigation, and more human studies in the clinical trial will lead to efficacious antioxidant-based therapies.

## Figures and Tables

**Table 1:** Mechanism involved in the production of reactive oxidants.

Oxidants (ROS)	Enzyme/Ion	Mechanism/Reaction	References
Superoxide radical (O_2_^−^)	NADPH oxidase	NADPH + 2O_2_ ➞ 2O_2_^−^ + NADP^+^ + H^+^	[15]
Hydrogen peroxide (H_2_O_2_)	SOD	2O_2_^−^ + 2H^+^ ➞ H_2_O_2_ + O_2_	[16]
Hydroxyl radical (OH^−^)	Fe^2+^	H_2_O_2_ ➞ OH^−^ + OH^.^	[17]
Hypochlorous acid (HOCl)	Myeloperoxidase	H_2_O_2_ + Cl^−^ + H^+^ ➞ HOCl + H_2_O	[18]
Peroxynitrite (OONO^−^)	NO^.^	NO^.^ + O_2_^−^ ➞ ONOO^−^	[19]
Nitric oxide (NO^.^)	NOS	L-Arginine + O_2_ ➞ L-Citrulline + NO	[20]

**Table 2:** List of antioxidants involved in sepsis.

Antioxidant		Location	Mechanism	References
Enzymatic	Superoxide dismutase	Cytoplasm, mitochondria, peroxisome, and chloroplast	O_2_^−^ to O_2_ and H_2_O_2_	[61,62]
	Catalase	Peroxisome and mitochondria	H_2_O_2_ to H_2_O and O_2_	[62,63]
	Glutathione peroxidase	Cytoplasm, mitochondria, and chloroplast	H_2_O_2_ to H_2_O	[64]
Non-enzymatic	Vitamin E (exogenous)	Cell membrane	Increases plasma NO, protects against oxidative damage induced-impaired vasorelaxation; prevents tumor initiation	[65]
	Vitamin C (exogenous)	Cytoplasm, mitochondria, peroxisome, and chloroplast	Decreases cellular oxidative damage, prevents tumor initiation; scavenging radical species; decreases pro-inflammatory cytokines; inhibition of NOx and inducible nitric oxide synthase	[49,66]
	Vitamin A (exogenous)	Chloroplasts	NF-κβ inhibition; 10% reduction of ROS by β-carotene; upregulation of Nrf2 expression	[67,68,69]
	N-Acetyl cysteine (NAC) (endogenous)	Administered orally or topically	Increases glutathione level in the human body; NAC acts as a methyl donor in the conversion of homocysteine to methionine	[70,71]
	Melatonin (endogenous)	Pineal gland	Reduces the free radical generation by increasing the activity of the electron transport chain	[72]

**Table 3:** Antioxidants investigated in sepsis and used in animal and clinical trials.

Vitamin	Animal Model	Tissue/ Cell	Outcomes	References
Vitamin A	Rats	Neutrophils	Reduces ROS generation by upregulating the SOD CAT, p22 and p47.	[159]
Vitamin A	Mice	Lung	Regulates expression of cytochrome P450	[160]
Vitamin A	Mice	Liver	Downregulation of NF-κβ associated VCAM-1, IL-1α, MCP-1 and IFN-γ	[161]
Vitamin A	Mice	Monoblasts	Decreased NF-κβ activity	[65]
Vitamin E	Human	Lung	Reduces the stroke in CVD	[162]
Vitamin E	Ex-vivo human samples	Neutrophils	Decreased O_2_^−^ plasma level	[163]
Vitamin E	Rat	Neutrophils	Induces an imbalance in hepatic vasoregulatory gene expression	[164]
Vitamin E	Human	Whole Body	Tends to decrease LPO	[165]
Vitamin C	Mice	Liver	Gene expression was changed	[166]
Vitamin C	Human	Endothelial cell	Improves endothelial function	[167]
Vitamin C	Sheep	Skin	Reduces inflammation	[113]
Vitamin C	Human	Whole Body	Increases its serum levels, which is associated with decreased levels of CRP, PCT, and NO_3_^−^/NO_2_^−^.	[165]
Glutathione	Rat	Mucosal tissue	Decreases glutathione concentration	[168]
Glutathione	Human	Plasma Cells	significantly decreased the peroxidative damage of patients with septic shock	[169]
SOD	Rat	Intestine	Increases sod concentration and decreased glutathione	[170]
SOD mimetic (M40401)	Rat	*E.coli* challenged animal, blood cells	Improves the vascular reactivity, reduced cytokine production, and mortality	[72]
SOD mimetic (MnIIITE-2-PyP5+)	Rat	Heart	Improves the vascular reactivity, reduced cytokine production, and mortality	[171]
CAT mimetic (EUK-207)	Mice	Brain cells	Reduces the level of lipid peroxidation and oxidized nucleic acids in brain cells	[172]
NAC	Rat	Hippocampus	Decreases the ROS activity as well as intracellular free Ca^2+^	[173]
NAC	Rat	Neural cell	Maintains the level of GSH, increases bioenergetics, and decreases oxidative damage	[174]
NAC	Human	Whole Body	Reduces LPO and improves antioxidant capacity	[165]
NAC	Human	Blood	Improves liver function	[137]
NAC	Human	Blood	Decreases hepatic lactate; increases liver perfusion and function	[175]
MitoQ	Rat	Lungs, heart, liver, gut, and kidney	Attenuation in the levels of biochemical markers of acute liver and renal dysfunction, maintenance of mitochondrial membrane potential in most organs.	[176]
MitoQ	Rat, Mouse	Heart	Restores mitochondrial function and reduces caspase activity	[177]
Hemigramicidin-TEMPO conjugates	Rat	Intestine	Improves the survival	[178]
Plastoquinone decylrhodamine 19 (SkQR1),	Rat	Kidney	Increases antioxidants and shows a nephroprotective role	[179]
MitoTEMPOL	Mouse	Diaphragm	Reduces sepsis-induced diaphragm dysfunction	[180]
SS31	Mouse		Restores myocardial morphological damage and suppresses inflammatory response	[181]
SS31	Mouse	Diaphragm	Reduces sepsis-induced diaphragm dysfunction, and maintains mitochondrial function	[182]

**Table 4:** Machine learning-based computational methods for prediction of antioxidant potential of proteins.

Server	Method	Sensitivity (%)	Specificity (%)	Accuracy (%)	Web-Server	Reference
AodPred	Support vector machine	75.09	74.48	74.79	http://lin.uestc.edu.cn/server/AntioxiPred (accessed on 1 January 2021)	[200]
SeqSVM	Support vector machine	---	---	89.46	---	[201]
AOPs-SVM	Support vector machine	68	98.5	94.2	http://server.malab.cn/AOPs-SVM/index.jsp (accessed on 1 January 2021)	[202]
Vote9	Support vector machine	65	99	94.1	---	[203]
SFS-SVM	Support vector machine	---	---	97.54	https://github.com/salman-khan-mrd/Antioxident_proteins (accessed on 1 January 2021)	[204]
AnOxPePred	Deep convolutional neural network	---	---	---	http://services.bioinformatics.dtu.dk/service.php?AnOxPePred-1.0 (accessed on 1 January 2021)	[205]

## Data Availability

Data included in the manuscript are available and can be shared if required.

## References

[1] Sherwood E.R., Toliver-Kinsky T. (2004). Mechanisms of the inflammatory response. Best Pract. Res. Clin. Anaesthesiol..

[2] Gotts J.E., Matthay M.A. (2016). Sepsis: Pathophysiology and clinical management. BMJ.

[3] Jean-Baptiste E. (2007). Cellular Mechanisms in Sepsis. J. Intensive Care Med..

[4] Cardoso L., Kauss I., Grion C., Cardoso L., Anami E., Nunes L., Ferreira G., Matsuo T., Bonametti A. (2009). Epidemiology of sepsis in a Brazilian teaching hospital. Crit. Care.

[5] Adhikari N.K.J., Fowler R.A., Bhagwanjee S., Rubenfeld G.D. (2010). Critical care and the global burden of critical illness in adults. Lancet.

[6] Neri A., Pezzotti P., Fazio C., Vacca P., D’Ancona F.P., Caporali M.G., Stefanelli P. (2015). Epidemiological and molecular characterization of invasive meningococcal disease in Italy, 2008/09-2012/13. PLoS ONE.

[7] Schulte W., Bernhagen J., Bucala R. (2013). Cytokines in sepsis: Potent immunoregulators and potential therapeutic targets—An updated view. Mediat. Inflamm..

[8] Russell J.A. (2006). Management of sepsis. N. Engl. J. Med..

[9] Cohen J. (2002). The immunopathogenesis of sepsis. Nature.

[10] Kumar S., Gupta E., Srivastava V.K., Kaushik S., Saxena J., Goyal L.K., Mehta S., Jyoti A. (2019). Nitrosative stress and cytokines are linked with the severity of sepsis and organ dysfunction. Br. J. Biomed. Sci..

[11] Azzi A. (2007). Oxidative stress: A dead end or a laboratory hypothesis?. Biochem. Biophys. Res. Commun..

[12] Prauchner C.A. (2017). Oxidative stress in sepsis: Pathophysiological implications justifying antioxidant co-therapy. Burns.

[13] Kehrer J.P. (2000). The Haber-Weiss reaction and mechanisms of toxicity. Toxicology.

[14] Qin H., Fan J., Mao S. (2020). Exploring the mechanism of the Fe(iii)-activated Fenton-like reaction based on a quantitative study. New J. Chem..

[15] Fridovich I. (1983). Superoxide Radical: An Endogenous Toxicant. Annu. Rev. Pharmacol. Toxicol..

[16] Lin S.-S., Gurol M.D. (1998). Catalytic Decomposition of Hydrogen Peroxide on Iron Oxide:  Kinetics, Mechanism, and Implications. Environ. Sci. Technol..

[17] Atkinson R. (1986). Kinetics and mechanisms of the gas-phase reactions of the hydroxyl radical with organic compounds under atmospheric conditions. Chem. Rev..

[18] Albrich J.M., McCarthy C.A., Hurst J.K. (1981). Biological reactivity of hypochlorous acid: Implications for microbicidal mechanisms of leukocyte myeloperoxidase. Proc. Natl. Acad. Sci. USA.

[19] Virág L., Szabó É., Gergely P., Szabó C. (2003). Peroxynitrite-induced cytotoxicity: Mechanism and opportunities for intervention. Toxicol. Lett..

[20] Griffith O.W., Stuehr D.J. (1995). Nitric Oxide Synthases: Properties and Catalytic Mechanism. Annu. Rev. Physiol..

[21] Sauer H., Wartenberg M., Hescheler J. (2001). Reactive Oxygen Species as Intracellular Messengers During Cell Growth and Differentiation. Cell. Physiol. Biochem..

[22] Di Meo S., Reed T.T., Venditti P., Victor V.M. (2016). Role of ROS and RNS Sources in Physiological and Pathological Conditions. Oxid. Med. Cell. Longev..

[23] Andrades M., Morina A., Spasić S., Spasojević I. (2011). Bench-to-bedside review: Sepsis—from the redox point of view. Crit. Care.

[24] Vanhoutte P.M. (2009). How We Learned to Say NO. Arterioscler. Thromb. Vasc. Biol..

[25] Murphy M.P. (2009). How mitochondria produce reactive oxygen species. Biochem. J..

[26] Guo R.-F., Ward P.A. (2007). Role of Oxidants in Lung Injury during Sepsis. Antioxid. Redox Signal..

[27] Barichello T., Fortunato J.J., Vitali Â.M., Feier G., Reinke A., Moreira J.C.F., Quevedo J., Dal-Pizzol F. (2006). Oxidative variables in the rat brain after sepsis induced by cecal ligation and perforation. Crit. Care Med..

[28] Park H.S., Jung H.Y., Park E.Y., Kim J., Lee W.J., Bae Y.S. (2004). Cutting edge: Direct interaction of TLR4 with NAD(P)H oxidase 4 isozyme is essential for lipopolysaccharide-induced production of reactive oxygen species and activation of NF-kappa B. J. Immunol..

[29] Gloire G., Legrand-Poels S., Piette J. (2006). NF-κB activation by reactive oxygen species: Fifteen years later. Biochem. Pharmacol..

[30] Kohchi C., Inagawa H., Nishizawa T., Soma G.-I. (2009). ROS and innate immunity. Anticancer Res..

[31] DeLeo F.R., Renee J., McCormick S., Nakamura M., Apicella M., Weiss J.P., Nauseef W.M. (1998). Neutrophils exposed to bacterial lipopolysaccharide upregulate NADPH oxidase assembly. J. Clin. Investig..

[32] Hughes G., Murphy M.P., Ledgerwood E.C. (2005). Mitochondrial reactive oxygen species regulate the temporal activation of nuclear factor kappaB to modulate tumour necrosis factor-induced apoptosis: Evidence from mitochondria-targeted antioxidants. Biochem. J..

[33] Förstermann U. (2008). Oxidative stress in vascular disease: Causes, defense mechanisms and potential therapies. Nat. Clin. Pract. Cardiovasc. Med..

[34] Kasahara S., Jhingran A., Dhingra S., Salem A., Cramer R.A., Hohl T.M. (2016). Role of Granulocyte-Macrophage Colony-Stimulating Factor Signaling in Regulating Neutrophil Antifungal Activity and the Oxidative Burst During Respiratory Fungal Challenge. J. Infect. Dis..

[35] Jyoti A., Singh A.K., Dubey M., Kumar S., Saluja R., Keshari R.S., Verma A., Chandra T., Kumar A., Bajpai V.K. (2014). Interaction of Inducible Nitric Oxide Synthase with Rac2 Regulates Reactive Oxygen and Nitrogen Species Generation in the Human Neutrophil Phagosomes: Implication in Microbial Killing. Antioxid. Redox Signal..

[36] Zingarelli B., Sheehan M., Wong H.R. (2003). Nuclear factor-κB as a therapeutic target in critical care medicine. Crit. Care Med..

[37] Sanlioglu S., Williams C.M., Samavati L., Butler N.S., Wang G., McCray P.B., Ritchie T.C., Hunninghake G.W., Zandi E., Engelhardt J.F. (2001). Lipopolysaccharide Induces Rac1-dependent Reactive Oxygen Species Formation and Coordinates Tumor Necrosis Factor-α Secretion through IKK Regulation of NF-κB. J. Biol. Chem..

[38] Blackwell T.S., Yull F.E., Chen C.-L., Venkatakrishnan A., Blackwell T.R., Hicks D.J., Lancaster L.H., Christman J.W., Kerr L.D. (2000). Multiorgan Nuclear Factor Kappa B Activation in a Transgenic Mouse Model of Systemic Inflammation. Am. J. Respir. Crit. Care Med..

[39] Böhrer H., Qiu F., Zimmermann T., Zhang Y., Jllmer T., Männel D., Böttiger B.W., Stern D.M., Waldherr R., Saeger H.D. (1997). Role of NFkappaB in the mortality of sepsis. J. Clin. Investig..

[40] Arnalich F., Garcia-Palomero E., López J., Jiménez M., Madero R., Renart J., Vázquez J.J., Montiel C. (2000). Predictive value of nuclear factor kappaB activity and plasma cytokine levels in patients with sepsis. Infect. Immun..

[41] Bertrand Y., Pincemail J., Hanique G., Denis B., Leenaerts L., Vankeerberghen L., Deby C. (1989). Differences in tocopherol-lipid ratios in ARDS and non-ARDS patients. Intensive Care Med..

[42] Mileva M., Dimitrova A., Krastev D., Alexandrova A., Tsvetanova E., Georgieva A., Galabov A. (2020). Oseltamivir and S-adenosyl-l-methionine combination as effective therapeutic strategy for suppression of oxidative damage in lung caused by influenza virus infection in mice. Drug Res..

[43] Metnitz P.G.H., Bartens C., Fischer M., Fridrich P., Steltzer H., Druml W. (1999). Antioxidant status in patients with acute respiratory distress syndrome. Intensive Care Med..

[44] Sakaguchi S., Furusawa S. (2006). Oxidative stress and septic shock: Metabolic aspects of oxygen-derived free radicals generated in the liver during endotoxemia. FEMS Immunol. Med. Microbiol..

[45] Lu X., Xue L., Sun W., Ye J., Zhu Z., Mei H. (2018). Identification of key pathogenic genes of sepsis based on the Gene Expression Omnibus database. Mol. Med. Rep..

[46] Galloway S.P., McMillan D.C., Sattar N. (2000). Effect of the inflammatory response on trace element and vitamin status. Ann. Clin. Biochem..

[47] Xing P.-C., An P., Hu G.-Y., Wang D.-L., Zhou M.-J. (2020). LncRNA MIAT Promotes Inflammation and Oxidative Stress in Sepsis-Induced Cardiac Injury by Targeting miR-330-5p/TRAF6/NF-κB Axis. Biochem. Genet..

[48] Langenberg C., Wan L., Egi M., May C.N., Bellomo R. (2007). Renal blood flow and function during recovery from experimental septic acute kidney injury. Intensive Care Med..

[49] Chand N., Sanyal A.J. (2007). Sepsis-induced cholestasis. Hepatology.

[50] Ciencewicki J., Trivedi S., Kleeberger S.R. (2008). Oxidants and the pathogenesis of lung diseases. J. Allergy Clin. Immunol..

[51] Wilson J.X., Wu F. (2012). Vitamin C in Sepsis. Subcell Biochem..

[52] Okado-Matsumoto A., Fridovich I. (2001). Subcellular distribution of superoxide dismutases (SOD) in rat liver: Cu, Zn-SOD in mitochondria. J. Biol. Chem..

[53] Ghio A.J., Suliman H.B., Carter J.D., Abushamaa A.M., Folz R.J. (2002). Overexpression of extracellular superoxide dismutase decreases lung injury after exposure to oil fly ash. Am. J. Physiol. Cell. Mol. Physiol..

[54] Berger M.M., Chioléro R.L. (2007). Antioxidant supplementation in sepsis and systemic inflammatory response syndrome. Crit. Care Med..

[55] Cruzat V.F., Bittencourt A., Scomazzon S.P., Leite J.S.M., de Bittencourt P.I.H., Tirapegui J. (2014). Oral free and dipeptide forms of glutamine supplementation attenuate oxidative stress and inflammation induced by endotoxemia. Nutrition.

[56] Zong Y., Zhang H. (2017). Amentoflavone prevents sepsis-associated acute lung injury through Nrf2-GCLc-mediated upregulation of glutathione. Acta Biochim. Pol..

[57] Li Z., Zhang X., Zhou H., Liu W., Li J. (2016). Exogenous S-nitrosoglutathione attenuates inflammatory response and intestinal epithelial barrier injury in endotoxemic rats. J. Trauma Acute Care Surg..

[58] Bailey J.D., Shaw A., McNeill E., Nicol T., Diotallevi M., Chuaiphichai S., Patel J., Hale A., Channon K.M., Crabtree M.J. (2020). Isolation and culture of murine bone marrow-derived macrophages for nitric oxide and redox biology. Nitric Oxide-Biol. Chem..

[59] Vico T.A., Marchini T., Ginart S., Lorenzetti M.A., Areán J.S.A., Calabró V., Garcés M., Ferrero M.C., Mazo T., D’Annunzio V. (2019). Mitochondrial bioenergetics links inflammation and cardiac contractility in endotoxemia. Basic Res. Cardiol..

[60] Cyr A., Chambers L., Waltz P.K., Whelan S.P., Kohut L., Carchman E., Dyer M., Luciano J., Kautza B., Gomez H.D. (2019). Endotoxin Engages Mitochondrial Quality Control via an iNOS-Reactive Oxygen Species Signaling Pathway in Hepatocytes. Oxid. Med. Cell. Longev..

[61] Yasui K., Baba A. (2006). Therapeutic potential of superoxide dismutase (SOD) for resolution of inflammation. Inflamm. Res..

[62] Kumar S., Gupta E., Kaushik S., Srivastava V.K., Mehta S.K., Jyoti A. (2018). Evaluation of oxidative stress and antioxidant status: Correlation with the severity of sepsis. Scand. J. Immunol..

[63] Shimozawa N., Zhang Z., Imamura A., Suzuki Y., Fujiki Y., Tsukamoto T., Osumi T., Aubourg P., Wanders R.J., Kondo N. (2000). Molecular Mechanism of Detectable Catalase-Containing Particles, Peroxisomes, in Fibroblasts from a PEX2-Defective Patient. Biochem. Biophys. Res. Commun..

[64] Kretz-Remy C., Mehlen P., Mirault M.E., Arrigo A.P. (1996). Inhibition of I kappa B-alpha phosphorylation and degradation and subsequent NF-kappa B activation by glutathione peroxidase overexpression. J. Cell Biol..

[65] Hon-Wing L., Vang M.J., Mavis R.D. (1981). The cooperative interaction between vitamin E and vitamin C in suppression of peroxidation of membrane phospholipids. Biochim. Biophys. Acta-Lipids Lipid Metab..

[66] Saánchez-Moreno C., Dashe J.F., Scott T., Thaler D., Folstein M.F., Martin A. (2004). Decreased Levels of Plasma Vitamin C and Increased Concentrations of Inflammatory and Oxidative Stress Markers After Stroke. Stroke.

[67] Austenaa L.M., Carlsen H., Hollung K., Blomhoff H.K., Blomhoff R. (2009). Retinoic acid dampens LPS-induced NF-κB activity: Results from human monoblasts and in vivo imaging of NF-κB reporter mice. J. Nutr. Biochem..

[68] Kim Y., Seo J.H., Kim H. (2011). β-Carotene and Lutein Inhibit Hydrogen Peroxide-Induced Activation of NF-κB and IL-8 Expression in Gastric Epithelial AGS Cells. J. Nutr. Sci. Vitaminol..

[69] Zhang X., Zhao W., Hu L., Zhao L., Huang J. (2011). Carotenoids inhibit proliferation and regulate expression of peroxisome proliferators-activated receptor gamma (PPARγ) in K562 cancer cells. Arch. Biochem. Biophys..

[70] Trabetti E. (2008). Homocysteine, MTHFR gene polymorphisms, and cardio-cerebrovascular risk. J. Appl. Genet..

[71] Griffith O.W., Meister A. (1979). Glutathione: Interorgan translocation, turnover, and metabolism. Proc. Natl. Acad. Sci. USA.

[72] Escames G., Lopez L.C., Tapias V., Utrilla P., Reiter R.J., Hitos A.B., Leon J., Rodriguez M.I., Acuna-Castroviejo D. (2006). Melatonin counteracts inducible mitochondrial nitric oxide synthase-dependent mitochondrial dysfunction in skeletal muscle of septic mice. J. Pineal Res..

[73] Macarthur H., Couri D.M., Wilken G.H., Westfall T.C., Lechner A.J., Matuschak G.M., Chen Z., Salvemini D. (2003). Modulation of serum cytokine levels by a novel superoxide dismutase mimetic, M40401, in an Escherichia coli model of septic shock: Correlation with preserved circulating catecholamines. Crit. Care Med..

[74] Nozik-Grayck E., Suliman H.B., Piantadosi C.A. (2005). Extracellular superoxide dismutase. Int. J. Biochem. Cell Biol..

[75] van Camp W., Inzé D., van Montagu M. (1997). The Regulation and Function of Tobacco Superoxide Dismutases. Free Radic. Biol. Med..

[76] Halliwell B. (1996). Antioxidants in Human Health and Disease. Annu. Rev. Nutr..

[77] Naito Y., Yoshikawa T., Ando T., Kishi A., Ueda S., Oyamada H., Kondo M. (1992). Changes in superoxide dismutase activity in the gastric mucosa of peptic ulcer patients. J. Clin. Gastroenterol..

[78] Kobayashi S.D., Voyich J.M., Somerville G.A., Braughton K.R., Malech H.L., Musser J.M., DeLeo F.R. (2003). An apoptosis-differentiation program in human polymorphonuclear leukocytes facilitates resolution of inflammation. J. Leukoc. Biol..

[79] Cuzzocrea S., Costantino G., Caputi A.P. (1998). Protective effect of melatonin on cellular energy depletion mediated by peroxynitrite and poly (ADP-ribose) synthetase activation in a non-septic shock model induced by zymosan in the rat. J. Pineal Res..

[80] Salvemini D., Riley D.P., Lennon P.J., Wang Z.-Q., Currie M.G., Macarthur H., Misko T.P. (1999). Protective effects of a superoxide dismutase mimetic and peroxynitrite decomposition catalysts in endotoxin-induced intestinal damage. Br. J. Pharmacol..

[81] Clares M.P., Blasco S., Inclán M., Agudo L.d., Verdejo B., Soriano C., Doménech A., Latorre J., García-España E. (2011). Manganese(ii) complexes of scorpiand-like azamacrocycles as MnSOD mimics. Chem. Commun..

[82] Clares M.P., Serena C., Blasco S., Nebot A., del Castillo L., Soriano C., Domènech A., Sánchez-Sánchez A.V., Soler-Calero L., Mullor J.L. (2015). Mn(II) complexes of scorpiand-like ligands. A model for the MnSOD active centre with high in vitro and in vivo activity. J. Inorg. Biochem..

[83] Serena C., Calvo E., Clares M.P., Diaz M.L., Chicote J.U., Beltrán-Debon R., Fontova R., Rodriguez A., García-España E., García-España A. (2015). Significant In Vivo Anti-Inflammatory Activity of Pytren4Q-Mn a Superoxide Dismutase 2 (SOD2) Mimetic Scorpiand-Like Mn (II) Complex. PLoS ONE.

[84] Coleman M., Brouillette M., Andresen N., Oberley-Deegan R., Martin J., Coleman M.C., Brouillette M.J., Andresen N.S., Oberley-Deegan R.E., Martin J.M. (2017). Differential Effects of Superoxide Dismutase Mimetics after Mechanical Overload of Articular Cartilage. Antioxidants.

[85] Schrader M., Fahimi H.D. (2006). Peroxisomes and oxidative stress. Biochim. Biophys. Acta-Mol. Cell Res..

[86] Ho Y.-S., Xiong Y., Ma W., Spector A., Ho D.S. (2004). Mice lacking catalase develop normally but show differential sensitivity to oxidant tissue injury. J. Biol. Chem..

[87] Siwale R.C., Oettinger C.W., Addo R., Siddig A., D’Souza M.J. (2009). The effect of intracellular delivery of catalase and antisense oligonucleotides to NF-κB using albumin microcapsules in the endotoxic shock model. J. Drug Target..

[88] Dong H.-P., Chunag I.-C., Wang D.-C., Huang L.-J., Lee C.-I., Tsai J.-H., Yang R.-C. (2010). Lipopolysaccharide-stimulated Leukocytes Contribute to Platelet Aggregative Dysfunction, Which is Attenuated by Catalase in Rats. Kaohsiung J. Med. Sci..

[89] Maksimenko A.V., Vavaeva A.V., Zvyagintseva M.A., Abramov A.A., Timoshin A.A., Vavaev A.V., Lakomkin V.L. (2016). [Protective action figurations for superoxide dismutase—Chondroitin sulfate—Catalase bienzyme conjugate after its medicative administration in endotoxin shock]. Biomed. Khim..

[90] Kanzok S.M., Fechner A., Bauer H., Ulschmid J.K., Müller H.M., Botella-Munoz J., Schneuwly S., Schirmer R.H., Becker K. (2001). Substitution of the thioredoxin system for glutathione reductase in Drosophila melanogaster. Science (80-).

[91] Bashir A., Perham R.N., Scrutton N.S., Berry A. (1995). Altering kinetic mechanism and enzyme stability by mutagenesis of the dimer interface of glutathione reductase. Biochem. J..

[92] Chang J.C., van der Hoeven L.H., Haddox C.H. (1978). Glutathione reductase in the red blood cells. Ann. Clin. Lab. Sci..

[93] García C., Moragón C., López-Fernández M.E. (1979). Frequency of Glutathione Reductase, Pyruvate Kinase and Glucose-6-Phosphate Dehydrogenase Deficiency in a Spanish Population. Hum. Hered..

[94] Redmond S.M., Joncourt F., Buser K., Ziemiecki A., Altermatt H.J., Fey M., Margison G., Cerny T. (1991). Assessment of P-glycoprotein, glutathione-based detoxifying enzymes and O6-alkylguanine-DNA alkyltransferase as potential indicators of constitutive drug resistance in human colorectal tumors. Cancer Res..

[95] Bounous G., Molson J.H. (2003). The antioxidant system. Anticancer Res..

[96] Şener G., Toklu H., Kapucu C., Ercan F., Erkanlı G., Kaçmaz A., Tilki M., Yeğen B.Ç. (2004). Melatonin Protects Against Oxidative Organ Injury in a Rat Model of Sepsis. Surg. Today.

[97] Samuvel D.J., Shunmugavel A., Singh A.K., Singh I., Khan M. (2016). S-Nitrosoglutathione ameliorates acute renal dysfunction in a rat model of lipopolysaccharide-induced sepsis. J. Pharm. Pharmacol..

[98] Khalili H. (2016). Ascorbic acid in septic shock. J. Res. Pharm. Pract..

[99] Kuhn S.-O., Meissner K., Mayes L.M., Bartels K. (2018). Vitamin C in sepsis. Curr. Opin. Anaesthesiol..

[100] Birben E., Sahiner U.M., Sackesen C., Erzurum S., Kalayci O. (2012). Oxidative Stress and Antioxidant Defense. World Allergy Organ. J..

[101] Venarucci D., Venarucci V., Vallese A., Battilà L., Casado A., de la Torre R., Fernandez M.E.L. (1999). Free radicals: Important cause of pathologies refer to ageing. Panminerva Med..

[102] Staal F.J.T., Roederer M., Raju P.A., Anderson M.T., Ela S.W., Herzenberg L.A., Herzenberg L.A. (1993). Antioxidants Inhibit Stimulation of HIV Transcription. AIDS Res. Hum. Retrovir..

[103] Victor V., Guayerbas N., Puerto M., Medina S., de la Fuente M. (2000). Ascorbic acid modulates in vitro the function of macrophages from mice with endotoxic shock. Immunopharmacology.

[104] Armour J., Tyml K., Lidington D., Wilson J.X. (2001). Ascorbate prevents microvascular dysfunction in the skeletal muscle of the septic rat. J. Appl. Physiol..

[105] Rojas C., Cadenas S., Herrero A., Méndez J., Barja G. (1996). Endotoxin depletes ascorbate in the guinea pig heart. Protective effects of vitamins C and E against oxidative stress. Life Sci..

[106] Galley H.F., Howdle P.D., Walker B.E., Webster N.R. (1997). The Effects of Intravenous Antioxidants in Patients with Septic Shock. Free Radic. Biol. Med..

[107] Quinlan G.J., Margarson M.P., Mumby S., Evans T.W., Gutteridge J.M. (1998). Administration of albumin to patients with sepsis syndrome: A possible beneficial role in plasma thiol repletion. Clin. Sci..

[108] Cárcamo J.M., Pedraza A., Bórquez-Ojeda O., Golde D.W. (2002). Vitamin C Suppresses TNFα-Induced NFκB Activation by Inhibiting IκBα Phosphorylation. Biochemistry.

[109] Wu F., Wilson J.X., Tyml K. (2003). Ascorbate inhibits iNOS expression and preserves vasoconstrictor responsiveness in skeletal muscle of septic mice. Am. J. Physiol. Integr. Comp. Physiol..

[110] Borrelli E., Roux-Lombard P., Grau G.E., Girardin E., Ricou B., Dayer J., Suter P.M. (1996). Plasma concentrations of cytokines, their soluble receptors, and antioxidant vitamins can predict the development of multiple organ failure in patients at risk. Crit. Care Med..

[111] Long C., Maull K., Krishnan R., Laws H., Geiger J., Borghesi L., Franks W., Lawson T., Sauberlich H. (2003). Ascorbic acid dynamics in the seriously ill and injured. J. Surg. Res..

[112] Fisher B.J., Kraskauskas D., Martin E.J., Farkas D., Puri P., Massey H.D., Idowu M.O., Brophy D.F., Voelkel N.F., Fowler A.A. (2014). Attenuation of Sepsis-Induced Organ Injury in Mice by Vitamin C. J. Parenter. Enter. Nutr..

[113] Tanaka H., Matsuda T., Miyagantani Y., Yukioka T., Matsuda H., Shimazaki S. (2000). Reduction of Resuscitation Fluid Volumes in Severely Burned Patients Using Ascorbic Acid Administration. Arch. Surg..

[114] Dubick M.A., Williams C., Elgjo G.I., Kramer G.C. (2005). High-dose Vitamin C infusion reduces fluid requirements in the resuscitation of burn-injured sheep. Shock.

[115] Fowler A.A., Syed A.A., Knowlson S., Sculthorpe R., Farthing D., DeWilde C., Farthing C.A., Larus T.L., Martin E., Brophy D.F. (2014). Phase I safety trial of intravenous ascorbic acid in patients with severe sepsis. J. Transl. Med..

[116] Mohammed B.M., Fisher B.J., Huynh Q.K., Wijesinghe D.S., Chalfant C.E., Brophy D.F., Fowler A.A., Natarajan R. (2014). Resolution of sterile inflammation: Role for vitamin C. Mediat. Inflamm..

[117] Traber M.G., Atkinson J. (2007). Vitamin E, antioxidant and nothing more. Free Radic. Biol. Med..

[118] Traber M.G., Packer L. (1995). Vitamin E: Beyond antioxidant function. Am. J. Clin. Nutr..

[119] Azzi A., Boscoboinik D., Chatelain E., Özer N.K., Stäuble B. (1993). d-α-tocopherol control of cell proliferation. Mol. Aspects Med..

[120] Goode H.F., Cowley H.C., Walker B.E., Howdle P.D., Webster N.R. (1995). Decreased antioxidant status and increased lipid peroxidation in patients with septic shock and secondary organ dysfunction. Crit. Care Med..

[121] Takeda K., Shimada Y., Amano M., Sakai T., Okada T., Yoshiya I. (1984). Plasma lipid peroxides and alpha-tocopherol in critically ill patients. Crit. Care Med..

[122] Yamamoto K., Niki E. (1988). Interaction of α-tocopherol with iron: Antioxidant and prooxidant effects of α-tocopherol in the oxidation of lipids in aqueous dispersions in the presence of iron. Biochim. Biophys. Acta-Lipids Lipid Metab..

[123] Peck M.D., Alexander J.W. (1991). Survival in Septic Guinea Pigs Is Influenced by Vitamin E, but Not by Vitamin C in Enteral Diets. J. Parenter. Enter. Nutr..

[124] Devaraj S., Li D., Jialal I. (1996). The effects of alpha tocopherol supplementation on monocyte function. Decreased lipid oxidation, interleukin 1 beta secretion, and monocyte adhesion to endothelium. J. Clin. Investig..

[125] Bulger E.M., Maier R.V. (2003). An argument for Vitamin E supplementation in the management of systemic inflammatory response syndrome. Shock.

[126] Atli M., Erikoglu M., Kaynak A., Esen H.H., Kurban S. (2012). The effects of selenium and vitamin E on lung tissue in rats with sepsis. Clin. Investig. Med..

[127] Koga H., Hagiwara S., Inomata M., Kono Y., Oyama Y., Kai S., Nishida T., Noguchi T. (2012). The New Vitamin E Derivative, ETS-GS, Protects Against Cecal Ligation and Puncture-Induced Systemic Inflammation in Rats. Inflammation.

[128] Gerster H. (1997). The potential role of lycopene for human health. J. Am. Coll. Nutr..

[129] Rao A.V., Rao L.G. (2007). Carotenoids and human health. Pharmacol. Res..

[130] Iyama T., Takasuga A., Azuma M. (1996). beta-Carotene accumulation in mouse tissues and a protective role against lipid peroxidation. Int. J. Vitam. Nutr. Res..

[131] Field C.J., Johnson I.R., Schley P.D. (2002). Nutrients and their role in host resistance to infection. J. Leukoc. Biol..

[132] Cox S.E., Arthur P., Kirkwood B.R., Yeboah-Antwi K., Riley E.M. (2006). Vitamin A supplementation increases ratios of proinflammatory to anti-inflammatory cytokine responses in pregnancy and lactation. Clin. Exp. Immunol..

[133] Bouayed J., Bohn T. (2010). Exogenous antioxidants--Double-edged swords in cellular redox state: Health beneficial effects at physiologic doses versus deleterious effects at high doses. Oxid. Med. Cell. Longev..

[134] Jang S.H., Lim J.W., Kim H. (2009). Beta-carotene inhibits Helicobacter pylori-induced expression of inducible nitric oxide synthase and cyclooxygenase-2 in human gastric epithelial AGS cells. J. Physiol. Pharmacol..

[135] Gawronski C.A., Gawronski K.M. (2016). Vitamin A Supplementation for Prevention of Bronchopulmonary Dysplasia. Ann. Pharmacother..

[136] Klassert T.E., Bräuer J., Hölzer M., Stock M., Riege K., Zubiría-Barrera C., Müller M.M., Rummler S., Skerka C., Marz M. (2017). Differential Effects of Vitamins A and D on the Transcriptional Landscape of Human Monocytes during Infection. Sci. Rep..

[137] Atkuri K.R., Mantovani J.J., Herzenberg L.A., Herzenberg L.A. (2007). N-Acetylcysteine—A safe antidote for cysteine/glutathione deficiency. Curr. Opin. Pharmacol..

[138] Rank N., Michel C., Haertel C., Lenhart A., Welte M., Meier-Hellmann A., Spies C. (2000). N-acetylcysteine increases liver blood flow and improves liver function in septic shock patients: Results of a prospective, randomized, double-blind study. Crit. Care Med..

[139] Zuin R., Palamidese A., Negrin R., Catozzo L., Scarda A., Balbinot M. (2005). High-Dose N-Acetylcysteine in??Patients with Exacerbations of??Chronic Obstructive Pulmonary Disease. Clin. Drug Investig..

[140] Pinkus R., Weiner L.M., Daniel V. (1996). Role of oxidants and antioxidants in the induction of AP-1, NF-kappaB, and glutathione S-transferase gene expression. J. Biol. Chem..

[141] Emet S., Memis D., Pamukçu Z. (2004). The influence of N-acetyl-L-cystein infusion on cytokine levels and gastric intramucosal pH during severe sepsis. Crit. Care..

[142] Spapen H.D., Diltoer M.W., Nguyen D.N., Hendrickx I., Huyghens L.P. (2005). Effects of N-acetylcysteine on Microalbuminuria and Organ Failure in Acute Severe Sepsis: Results of a Pilot Study. Chest.

[143] Çağlıkülekci M., Pata C., Apa D.D., Dirlik M., Tamer L., Yaylak F., Kanik A., Aydin S. (2004). The effect of N-acetylcysteine (NAC) on liver and renal tissue inducible nitric oxide synthase (iNOS) and tissue lipid peroxidation in obstructive jaundice stimulated by lipopolysaccharide (LPS). Pharmacol. Res..

[144] Najafi A., Mojtahedzadeh M., Ahmadi K., Abdollahi M., Mousavi M., Chelkeba L., Najmeddin F., Ahmadi A. (2014). The immunological benefit of higher dose N-acetyl cysteine following mechanical ventilation in critically ill patients. DARU J. Pharm. Sci..

[145] Gomes B.Q., da Silva L., Gomes A.Q., Moreira D., Dolabela M., Santos R., Green M., Carvalho E., Percário S. (2015). N-acetyl cysteine and mushroom Agaricus sylvaticus supplementation decreased parasitaemia and pulmonary oxidative stress in a mice model of malaria. Malar. J..

[146] Ergin B., Guerci P., Zafrani L., Nocken F., Kandil A., Gurel-Gurevin E., Demirci-Tansel C., Ince C. (2016). Effects of N-acetylcysteine (NAC) supplementation in resuscitation fluids on renal microcirculatory oxygenation, inflammation, and function in a rat model of endotoxemia. Intensive Care Med. Exp..

[147] Visvanathan V. (2013). N-acetylcysteine for sepsis and systemic inflammatory response in adults. Crit. Care Nurse..

[148] Brzezinski A. (1997). Melatonin in Humans. N. Engl. J. Med..

[149] Tan D.-X., Manchester L.C., Reiter R.J., Plummer B.F., Limson J., Weintraub S.T., Qi W. (2000). Melatonin directly scavenges hydrogen peroxide: A potentially new metabolic pathway of melatonin biotransformation. Free Radic. Biol. Med..

[150] Reiter R.J., Tan D.-X., Korkmaz A. (2009). The circadian melatonin rhythm and its modulation: Possible impact on hypertension. J. Hypertens..

[151] Cuzzocrea S., Mazzon E., Dugo L., Caputi A.P., Aston K., Riley D.P., Salvemini D. (2001). Protective effects of a new stable, highly active SOD mimetic, M40401 in splanchnic artery occlusion and reperfusion. Br. J. Pharmacol..

[152] López L.C., Escames G., Tapias V., Utrilla P., León J., Acuña-Castroviejo D. (2006). Identification of an inducible nitric oxide synthase in diaphragm mitochondria from septic mice: Its relation with mitochondrial dysfunction and prevention by melatonin. Int. J. Biochem. Cell Biol..

[153] Escames G., León J., Macías M., Khaldy H., Acuña-Castroviejo D. (2003). Melatonin counteracts lipopolysaccharide-induced expression and activity of mitochondrial nitric oxide synthase in rats. FASEB J..

[154] Ozkok E., Yorulmaz H., Ates G., Aksu A., Balkis N., Şahin Ö., Tamer S. (2016). Amelioration of energy metabolism by melatonin in skeletal muscle of rats with LPS induced endotoxemia. Physiol. Res..

[155] Plessis S.S.d., Hagenaar K., Lampiao F. (2010). The in vitro effects of melatonin on human sperm function and its scavenging activities on NO and ROS. Andrologia.

[156] Zavodnik I.B., Domanski A.V., Lapshina E.A., Bryszewska M., Reiter R.J. (2006). Melatonin directly scavenges free radicals generated in red blood cells and a cell-free system: Chemiluminescence measurements and theoretical calculations. Life Sci..

[157] Hardeland R., Cardinali D.P., Brown G.M., Pandi-Perumal S.R. (2015). Melatonin and brain inflammaging. Prog. Neurobiol..

[158] Hu W., Deng C., Ma Z., Wang D., Fan C., Li T., Di S., Gong B., Reiter R.J., Yang Y. (2017). Utilizing melatonin to combat bacterial infections and septic injury. Br. J. Pharmacol..

[159] El-Gendy F.M., El-Hawy M.A., Hassan M.G. (2018). Beneficial effect of melatonin in the treatment of neonatal sepsis. J. Matern. Neonatal Med..

[160] Júnior J.V.R., Araújo G.R., Pádua B.D.C., Magalhães C.L.d., Chaves M.M., Pedrosa M.L., Silva M.E., Costa D.C. (2012). Annatto extract and β-carotene enhances antioxidant status and regulate gene expression in neutrophils of diabetic rats. Free Radic. Res..

[161] Aung H.H., Vasu V.T., Valacchi G., Corbacho A.M., Kota R.S., Lim Y., Obermueller-Jevic U.C., Packer L., Cross C.E., Gohil K. (2009). Effects of dietary carotenoids on mouse lung genomic profiles and their modulatory effects on short-term cigarette smoke exposures. Genes Nutr..

[162] Harari A., Harats D., Marko D., Cohen H., Barshack I., Kamari Y., Gonen A., Gerber Y., Ben-Amotz A., Shaish A. (2008). A 9-cis β-Carotene–Enriched Diet Inhibits Atherogenesis and Fatty Liver Formation in LDL Receptor Knockout Mice. J. Nutr..

[163] Milman U., Blum S., Shapira C., Aronson D., Miller-Lotan R., Anbinder Y., Alshiek J., Bennett L., Kostenko M., Landau M. (2008). Vitamin E Supplementation Reduces Cardiovascular Events in a Subgroup of Middle-Aged Individuals with Both Type 2 Diabetes Mellitus and the Haptoglobin 2-2 Genotype. Arterioscler. Thromb. Vasc. Biol..

[164] Durant R., Klouche K., Delbosc S., Morena M., Amigues L., Beraud J.J., Canaud B., Cristol J.P. (2004). Superoxide anion overproduction in sepsis: Effects of vitamin E and simvastatin. Shock.

[165] Kim J.Y., Lee S.M. (2005). Effect of alpha-tocopherol on the expression of hepatic vascular stress genes in response to sepsis. J. Toxicol. Environ. Health-Part A.

[166] Aisa-Alvarez A., Gamboa R., Guarner-Lans V., Soto M. (2020). A Randomized clinical trial of antioxidant therapy in patients with septic shock. Reference study to propose adjuvant therapy in patients with critical organic damage by COVID-19. EuropePMC.

[167] Jun H.-J., Kim S., Dawson K., Choi D.-W., Kim J.-S., Rodriguez R.L., Lee S.-J. (2011). Effects of Acute Oral Administration of Vitamin C on the Mouse Liver Transcriptome. J. Med. Food.

[168] May J.M., Harrison F.E. (2013). Role of Vitamin C in the Function of the Vascular Endothelium. Antioxid. Redox Signal..

[169] Ardite E., Sans M., Panés J., Romero F.J., Piqué J.M., Fernández-Checa J.C. (2000). Replenishment of Glutathione Levels Improves Mucosal Function in Experimental Acute Colitis. Lab. Investig..

[170] Ortolani O., Conti A., de Gaudio A.R., Moraldi E., Cantini Q., Novelli G. (2000). The effect of glutathione and N-acetylcysteine on lipoperoxidative damage in patients with early septic shock. Am. J. Respir. Crit. Care Med..

[171] Nieto N., Torres M.I., Fernández M.I., Girón M.D., Ríos A., Suárez M.D., Gil A. (2000). Experimental Ulcerative Colitis Impairs Antioxidant Defense System in Rat Intestine. Dig. Dis. Sci..

[172] Spasojević I., Chen Y., Noel T.J., Yu Y., Cole M.P., Zhang L., Zhao Y., Clair D.K.S., Batinić-Haberle I. (2007). Mn porphyrin-based superoxide dismutase (SOD) mimic, MnIIITE-2-PyP5+, targets mouse heart mitochondria. Free Radic. Biol. Med..

[173] Clausen A., Xu X., Bi X., Baudry M. (2012). Effects of the Superoxide Dismutase/Catalase Mimetic EUK-207 in a Mouse Model of Alzheimer’s Disease: Protection Against and Interruption of Progression of Amyloid and Tau Pathology and Cognitive Decline. J. Alzheimer’s Dis..

[174] Nazıroğlu M., Şenol N., Ghazizadeh V., Yürüker V. (2014). Neuroprotection Induced by N-acetylcysteine and Selenium Against Traumatic Brain Injury-Induced Apoptosis and Calcium Entry in Hippocampus of Rat. Cell. Mol. Neurobiol..

[175] Pandya J.D., Readnower R.D., Patel S.P., Yonutas H.M., Pauly J.R., Goldstein G.A., Rabchevsky A.G., Sullivan P.G. (2014). N-acetylcysteine amide confers neuroprotection, improves bioenergetics and behavioral outcome following TBI. Exp. Neurol..

[176] Hein O.V., Ohring R., Schilling A., Oellerich M., Armstrong V.W., Kox W.J., Spies C. (2004). N-acetylcysteine decreases lactate signal intensities in liver tissue and improves liver function in septic shock patients, as shown by magnetic resonance spectroscopy: Extended case report. Crit. Care.

[177] Lowes D.A., Thottakam B.M.V., Webster N.R., Murphy M.P., Galley H.F. (2008). The mitochondria-targeted antioxidant MitoQ protects against organ damage in a lipopolysaccharide-peptidoglycan model of sepsis. Free Radic. Biol. Med..

[178] Supinski G.S., Murphy M.P., Callahan L.A. (2009). MitoQ administration prevents endotoxin-induced cardiac dysfunction. Am. J. Physiol.-Regul. Integr. Comp. Physiol..

[179] Macias C.A., Chiao J.W., Xiao J., Arora D.S., Tyurina Y.Y., Delude R.L., Wipf P., Kagan V.E., Fink M.P. (2007). Treatment with a novel hemigramicidin-TEMPO conjugate prolongs survival in a rat model of lethal hemorrhagic shock. Ann. Surg..

[180] Plotnikov E.Y., Pevzner I.B., Zorova L.D., Chernikov V.P., Prusov A.N., Kireev I.I., Silachev D.N., Skulachev V.P., Zorov D.B. (2019). Mitochondrial damage and mitochondria-targeted antioxidant protection in LPS-induced acute kidney injury. Antioxidants.

[181] Supinski G.S., Wang L., Schroder E.A., Callahan L.A.P. (2020). MitoTEMPOL, a mitochondrial targeted antioxidant, prevents sepsis-induced diaphragm dysfunction. Am. J. Physiol.-Lung Cell. Mol. Physiol..

[182] Liu Y., Yang W., Sun X., Xie L., Yang Y., Sang M., Jiao R. (2019). SS31 Ameliorates Sepsis-Induced Heart Injury by Inhibiting Oxidative Stress and Inflammation. Inflammation.

[183] Supinski G.S., Wang L., Schroder E.A., Callahan L.A.P. (2020). SS31, a mitochondrially targeted antioxidant, prevents sepsis-induced reductions in diaphragm strength and endurance. J. Appl. Physiol..

[184] Zhang S., Zhou Q., Li Y., Zhang Y., Wu Y. (2020). MitoQ Modulates Lipopolysaccharide-Induced Intestinal Barrier Dysfunction via Regulating Nrf2 Signaling. Mediat. Inflamm..

[185] Galkin I.I., Pletjushkina O.Y., Zinovkin R.A., Zakharova V.V., Chernyak B.V., Popova E.N. (2016). Mitochondria-targeted antioxidant SkQR1 reduces TNF-induced endothelial permeability in vitro. Biochemistry.

[186] Shan B., Li J.-Y., Liu Y.-J., Tang X.-B., Zhou Z., Luo L.-X. (2020). LncRNA H19 Inhibits the Progression of Sepsis-Induced Myocardial Injury via Regulation of the miR-93-5p/SORBS2 Axis. Inflammation.

[187] Liu Z.-Q. (2014). Antioxidants may not always be beneficial to health. Nutrition.

[188] Fang J.C., Kinlay S., Beltrame J., Hikiti H., Wainstein M., Behrendt D., Suh J., Frei B., Mudge G.H., Selwyn A. (2002). Effect of vitamins C and E on progression of transplant-associated arteriosclerosis: A randomised trial. Lancet.

[189] Pironi L., Guidetti M., Zolezzi C., Fasano M.C., Paganelli F., Merli C., Bersani G., Pizzoferrato A., Miglioli M. (2003). Peroxidation potential of lipid emulsions after compounding in all-in-one solutions. Nutrition.

[190] Dyer A., Elliott P., Stamler J., Chan Q., Ueshima H., Zhou B. (2003). Dietary intake in male and female smokers, ex-smokers and never smokers: The INTERMAP Study. J. Hum. Hypertens..

[191] Miller E.R., Pastor-Barriuso R., Dalal D., Riemersma R.A., Appel L.J., Guallar E. (2005). Meta-Analysis: High-Dosage Vitamin E Supplementation May Increase All-Cause Mortality. Ann. Intern. Med..

[192] Mitra S., Deshmukh A., Sachdeva R., Lu J., Mehta J.L. (2011). Oxidized Low-Density Lipoprotein and Atherosclerosis Implications in Antioxidant Therapy. Am. J. Med. Sci..

[193] Ramos R., Martínez-Castelao A. (2008). Lipoperoxidation and hemodialysis. Metabolism.

[194] Agarwal A., Majzoub A. (2017). Laboratory tests for oxidative stress. Indian J. Urol..

[195] Waldbaum S., Patel M. (2010). Mitochondrial dysfunction and oxidative stress: A contributing link to acquired epilepsy?. J. Bioenerg. Biomembr..

[196] Baba S., Osakabe N., Kato Y., Natsume M., Yasuda A., Kido T., Fukuda K., Muto Y., Kondo K. (2007). Continuous intake of polyphenolic compounds containing cocoa powder reduces LDL oxidative susceptibility and has beneficial effects on plasma HDL-cholesterol concentrations in humans. Am. J. Clin. Nutr..

[197] Havsteen B.H. (2002). The biochemistry and medical significance of the flavonoids. Pharmacol. Ther..

[198] Procházková D., Boušová I., Wilhelmová N. (2011). Antioxidant and prooxidant properties of flavonoids. Fitoterapia.

[199] Galati G., O’Brien P.J. (2004). Potential toxicity of flavonoids and other dietary phenolics: Significance for their chemopreventive and anticancer properties. Free Radic. Biol. Med..

[200] Feng P., Chen W., Lin H. (2016). Identifying Antioxidant Proteins by Using Optimal Dipeptide Compositions. Interdiscip. Sci. Comput. Life Sci..

[201] Xu L., Liang G., Shi S., Liao C. (2018). SeqSVM: A Sequence-Based Support Vector Machine Method for Identifying Antioxidant Proteins. Int. J. Mol. Sci..

[202] Meng C., Jin S., Wang L., Guo F., Zou Q. (2019). AOPs-SVM: A Sequence-Based Classifier of Antioxidant Proteins Using a Support Vector Machine. Front. Bioeng. Biotechnol..

[203] Li X., Tang Q., Tang H., Chen W. (2020). Identifying Antioxidant Proteins by Combining Multiple Methods. Front. Bioeng. Biotechnol..

[204] Ahmad A., Akbar S., Hayat M., Ali F., Khan S., Sohail M. (2020). Identification of antioxidant proteins using a discriminative intelligent model of k-spaced amino acid pairs based descriptors incorporating with ensemble feature selection. Biocybern. Biomed. Eng..

[205] Olsen T.H., Yesiltas B., Marin F.I., Pertseva M., García-Moreno P.J., Gregersen S., Overgaard M.T., Jacobsen C., Lund O., Hansen E.B. (2020). AnOxPePred: Using deep learning for the prediction of antioxidative properties of peptides. Sci. Rep..

